# Behavioral disruption in honey bees (*Apis mellifera*) exposed to isolated and combined insecticides

**DOI:** 10.1007/s10646-026-03095-8

**Published:** 2026-04-28

**Authors:** Vagner Luiz Graeff-Filho, Luiz Ernesto Costa-Schmidt, Stéphane Ramos Idalgo, Felipe Diehl, Cristiano Agra Iserhard

**Affiliations:** 1 Programa de Pós-Graduação em Fitossanidade, Universidade Federal de Pelotas (UFPel), Pelotas, Rio Grande do Sul State Brasil; 2 Programa de Pós-Graduação em Biodiversidade Animal, Universidade Federal de Pelotas (UFPel), Pelotas, Rio Grande do Sul State Brasil; 3 Departamento de Ecologia, Zoologia e Genética, Universidade Federal de Pelotas (UFPel), Pelotas, Rio Grande do Sul State Brasil

**Keywords:** Grooming, Locomotion, Neonicotinoids, Pyrethroids, Sublethal dose

## Abstract

The global decline of pollinators threatens ecosystem stability and food security. Bees are particularly affected by agricultural insecticides, with neonicotinoids and pyrethroids being among the most widely used classes. While their individual sublethal effects are documented, the consequences of combined exposures remain poorly understood. This study evaluated the isolated and combined effects of field-realistic oral doses of Imidacloprid (0.5 ng/bee) and Deltamethrin (2 ng/bee) on Africanized honey bees (*Apis mellifera*) of southern Brazil. We quantified grooming, wing fanning, feeding and locomotion (walking time, distance and speed) under laboratory conditions. Imidacloprid caused a strong elevation in grooming behavior (8.64-fold) and reduced both walking distance (-308 cm) and speed (-1.04 cm·s⁻¹). Deltamethrin increased grooming to a lesser extent (2.96-fold) without affecting any locomotion capability. Combined exposure resulted in non-additive effects, with grooming activity reaching levels similar to those of Imidacloprid alone (7.79-fold). However, unlike Imidacloprid, the mixture caused no locomotor deficits, which contrasts with the additive or synergistic interactions commonly reported for insecticide mixtures. No treatment affected syrup consumption or mortality over a 24 h period. These results demonstrate that neonicotinoids and pyrethroids disrupt honey bee behavior through distinct pathways and that mixture effects cannot be predicted from single-compound responses. Incorporating behavioral endpoints and mixture toxicology into risk assessments is essential for improving pesticide-pollinator dynamics and protection strategies.

**Supplementary Information:**

The online version contains supplementary material available at 10.1007/s10646-026-03095-8.

## Introduction

Bees are the most important group among pollinators, visiting more than 90% of the leading 107 global crops (Klein et al. 2007), reinforcing the importance of their contribution to ecosystems and agriculture (Gallai et al. 2009). Paradoxically, some agricultural practices, such as the use of pesticides, linked with other anthropogenic impacts such as deforestation, contribute to the decline of this vital ecosystem service (Potts et al. 2010; Goulson et al. 2015; Dicks et al. 2021). Thus, global pollination service is at risk due to the decline of insect populations, compromising the stability of ecosystems and food security (IPBES 2016; Hallmann et al. 2017; Murphy et al. 2022). Understanding how these practices affect pollinators is therefore essential for developing effective mitigation strategies.

Bees are exposed to pesticides through direct contact during or after application, as well as through the ingestion of contaminated nectar, pollen, or water (Botías et al. 2015; Zioga et al. 2020). For social species such as honey bees, exposure also occurs via contact with contaminated nest products (Calatayud-Vernich et al. 2018). Many insecticides are systemic and translocate within plant tissues, contaminating floral resources and increasing pollinator exposure (Bonmatin et al. 2015; Simon-Delso et al. 2015).

Although the negative impacts of insecticides on bees are well documented, the consequences of simultaneous exposure to multiple compounds remain poorly understood (Blacquière et al. 2012; Gill et al. 2012; Benuszak et al. 2017; Bartling et al. 2024). This knowledge gap limits risk assessment accuracy and the development of effective regulatory policies (Christen et al. 2017; Tosi et al. 2022). Traditional toxicity assessments rely heavily on LD50 values derived from acute laboratory tests, which estimate mortality within 24–48 h but fail to capture sublethal impairments affecting behavior, cognition, and physiology (Mullin et al. 2010; Wu et al. 2011). These sublethal effects, while not immediately fatal, can significantly compromise colony fitness by impairing traits linked to survival and reproduction (Mullin et al. 2010; Wu et al. 2011), highlighting the need for sensitive and integrative assessment tools (Barascou et al. 2021).

Among these compounds, neonicotinoids are of particular concern, accounting for approximately 25% of the global insecticide market, with Imidacloprid being one of the most widely used insecticides worldwide and presenting well-documented deleterious effects on pollinators (Thompson et al. 2020; Klingelhöfer et al. 2022; Tosi et al. 2022; Bartling et al. 2024), while remaining authorized in over 120 countries and used on more than 140 crops (Klingelhöfer et al. 2022). Imidacloprid acts as an agonist of nicotinic acetylcholine receptors, causing persistent neuronal excitation (Tomizawa and Casida 2005). This compound is among the most extensively studied insecticides due to its well-documented impairment of cognitive functions, including olfactory, visual, and aversive learning and memory (Zhang and Nieh 2015; DesJardins et al. 2023; Graeff-Filho et al. 2025 ; Rükün et al. 2025). Additionally, Imidacloprid negatively affects gustatory sensitivity (Eiri and Nieh 2012), gene expression (Zhang et al. 2022), locomotion (walking and flight), and complex social behaviors such as waggle dancing and grooming (Williamson et al. 2014; Alkassab and Kirchner 2018).

Complementary, Deltamethrin is a pyrethroid insecticide that disrupts the central nervous system by modulating voltage-gated sodium channels (Narahashi 1996; Soderlund et al. 2012; Field et al. 2017), leading to alterations in honey bees, such as locomotion, learning, feeding, gene expression, and self-cleaning activities (Vandame et al. 1995; Decourtye et al. 2004; Charreton et al. 2015; Thany et al. 2015). In agricultural landscapes, Deltamethrin frequently co-occurs with other insecticides, including Imidacloprid, resulting in realistic exposure scenarios involving multiple compounds (Wen et al. 2021; Tosi et al. 2022). Although both insecticides are known to individually compromise honey bee performance in multiple tasks, ultimately reducing foraging efficiency and survival (Ramirez-Romero et al. 2005; Henry et al. 2012; Baracchi 2019), the potential interaction and cumulative effects of co-exposure to these distinct chemical classes remain largely unexplored.

The assessment of behavioral endpoints emerges as a vital strategy to unravel these complex interactions. Behavioral analysis, particularly movement-related endpoints, provides a powerful framework for detecting sublethal insecticide effects (Bartling et al. 2024). Changes in navigation, exploration, locomotion, and grooming reflect disruptions in neural and muscular function that directly affect pollination efficiency and colony performance (Thompson 2003; Teeters et al. 2012). Motor performance is fundamental for in-hive activities and foraging, as bees must efficiently move, manipulate flowers, and perform learning-dependent tasks (Moritz and Southwick 1992; Abramson et al. 2016; Cabirol et al. 2018). Grooming behavior, which involves the removal of debris or parasites from the body, is particularly relevant as it is highly sensitive to pesticide exposure and plays a key role in colony hygiene (Boecking and Spivak 1999; Russo et al. 2020).

Monitoring bee movement therefore allows the detection of physiological and behavioral impairments that may remain undetected by mortality-based assays (Teeters et al. 2012; Tosi et al. 2022). The use of automated video-tracking tools enhances the sensitivity and reproducibility of these analyses, enabling the quantification of subtle alterations under field-realistic exposure conditions (Morfin et al. 2020; Siefert et al. 2021). In this study, we evaluated the effects of oral exposure to Imidacloprid and Deltamethrin, alone and in combination, on honey bee behavior, hypothesizing that sublethal doses would impair locomotion and grooming without causing acute mortality, and that combined exposure to both insecticides would exacerbate these effects due to complementary neurotoxic mechanisms.

By focusing on sublethal behavioral endpoints under realistic exposure scenarios, this work contributes to a more ecologically relevant assessment of insecticide risks. Because bees are rarely exposed to single compounds, evaluating mixture effects is essential for accurate risk prediction (Rundlöf et al. 2015; Tosi et al. 2022). Our findings support the inclusion of behavioral toxicity and multi-compound exposure in regulatory risk assessment frameworks and demonstrate the value of video-based behavioral analysis as a tool for improving pesticide evaluation and promoting more sustainable agricultural practices (Goulson et al. 2015; Dicks et al. 2016; IPBES 2016).

## Materials and methods

### Honey bee sampling

Mixed-age, African-European honey bees (*Apis mellifera* L.) were collected in the experimental apiary of Centro Agropecuário da Palma of Universidade Federal de Pelotas (UFPel) (Capão do Leão municipality, Rio Grande do Sul State, Brazil; 31.802416°S, 52.508107°W). We gathered all samples from three colonies during the summer, following the protocol developed by Scheiner et al. (2015), in which we collected around 100 bees per hive. Each colony had a natural queen succession, without presenting symptoms of disease or acaricide treatments. We captured the foraging bees at the hive entrances using transparent plastic containers and immediately took them to the *Laboratório de Ecologia de Lepidoptera* (LELep) located at UFPel. We kept each bee for 30 min for adaptation in individualized plastic containers with a hole to allow airflow (container volume: 2 mL) in an acclimatized room (25° C, 65% relative humidity (RH)) before the insecticide administration.

### Insecticide administration

The insecticide selection criteria were based on its widespread use in farming areas worldwide, including large quantities sold annually, and the recognized deleterious effects on *Bombus* spp. and *Apis mellifera* bees. We used the following commercial insecticides: Imidacloprid Nortox (480 g a.i/L) (IMI) of the neonicotinoid group, and Deltamethrin Decis 25 EC^®^ (25 g a.i /L) (DEL) of the pyrethroid group. Doses were defined at a field-relevant scale, based on surveys of nectar (DEL: 23.89–43.01 ng/g; IMI: 0.01–6588 ng/g) and pollen (DEL: 389.72–610.78 ng/g; IMI: 0.01–150 ng/g) contamination (Rortais et al. 2005; Wood and Goulson 2017; Zioga et al. 2020; Wen et al. 2021) and the estimated daily consumption of nectar (44 mg) and pollen (3.9 mg) *per* bee (Rortais et al. 2005).

We divided bees into four groups of insecticide treatments (15 bees per treatment), dissolved by successive dilutions in sucrose solution carrier (1.7 M) to achieve the final doses: Control (only sugar syrup); DEL: 2 ng a.i/bee (Oral LD_50_ 70 ng a.i/bee); IMI: 0.5 ng a.i/bee (Oral LD_50_ 3.7 ng a.i/bee); COM: combination of DEL 2 ng a.i/bee + IMI 0.5 ng a.i/bee (Oral LD_50_ unknown). We administered the insecticides via oral exposure by delivering 2 µL of contaminated sucrose solution directly to the bee’s mouthparts using a micropipette. Bees that failed to ingest the sucrose solution were discarded and excluded from the analyses. After insecticide exposures, bees rested for 30 min before the observational tests to metabolize the substances and habituate to the laboratory setup conditions.

The experimental trials were conducted in a staggered sequential design. Bees were organized into blocks of four individuals (one per treatment group, 4 treatments per block) exposed to insecticides. This procedure ensured a standardized 30-minute metabolization period for each individual before their behavioral assay. We performed 15 consecutive trials, totaling 60 bees divided into 15 bees per treatment.

### Observational setup and image analysis

We video recorded all trials using a proper setup to minimize the effect of observer presence and the surrounding environment. For this, we carried out the recordings in a room covered with black fabric to avoid external light interference, at a constant temperature of 25 °C. This setup allowed us to describe and quantify the bees’ investment in locomotion and grooming (Thompson 2003; Scheiner et al. 2013) using both manual observation and software tracking.

**Fig. 1:**
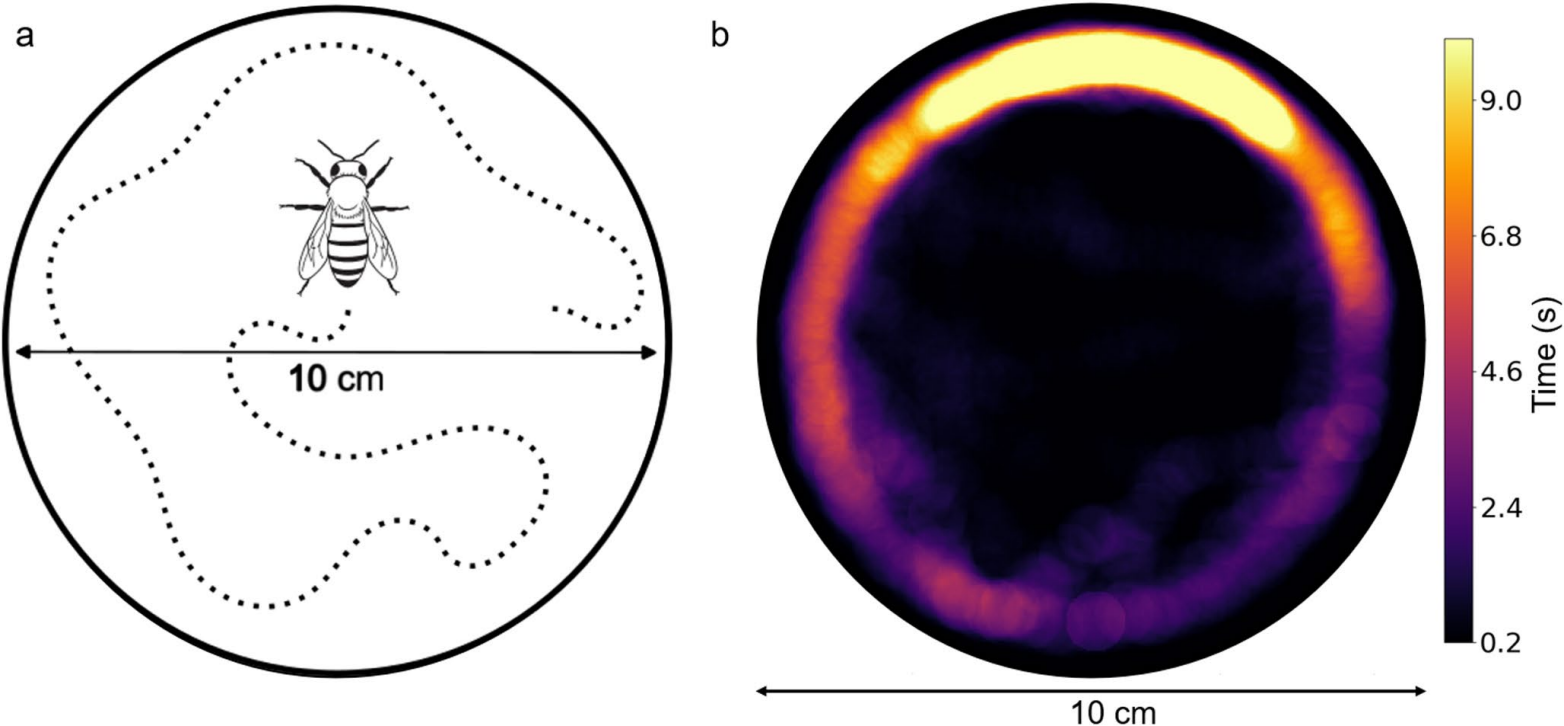
**a** Scheme of the test arena consisting of a Petri dish (10 cm diameter), allowing the bee to move freely, limited on the sides and top by a glass lid, enabling observation and high-quality video recording. The dotted line represents a hypothetical trajectory adopted by the bee within the arena. **b** Representative heat map of the edge-exploring pattern of a honey bee within the arena. The plot is based on the trajectory of a control-group individual

The trial arenas consisted of a glass Petri dish (10 cm diameter; 1 cm in height), with a white sheet of paper (75 g/m²) at the bottom to enhance contrast (Fig. 1A). We replaced the entire arena after each trial. The recording setup consisted of a camera (Logitech C920 Full Hd 30fps) and a white LED lamp of six lumens, both fixed at a height of 50 cm above the center of the arena.

We introduced each bee into the arena without using anesthesia to prevent stress associated with the procedure, by exploring the bee’s positive phototactic behavior. This procedure consisted of positioning the arena above a hole through which the bee could actively ascend into the arena. As soon as the bee entered the arena, we gently moved it onto the sheet, sealing the entrance, and allowing the bee to explore the arena (Fig. 1B). We recorded the bees’ reactions for five minutes in blocks of four independent arenas, one for each experimental treatment (Control, DEL, IMI, COM). After each trial, the arena positions were randomized, and both the arenas and the sheet of paper were replaced.

### Behavioral activities

We applied two complementary methods for data recording. First, we used a tracking software (AnimalTA, Version 3.2.2; Chiara and Kim 2023) to track the bees’ individual paths and obtain average walking speed, distance traveled, and the proportion of time moving. Second, we manually recorded the bee’s total time spent grooming, resting, wing fanning, and lying on its back using a digital clock to count the time spent on each behavior (Table 1).

**Table 1:** Behaviors of bees measured in the assay

Behavior	Description	Unit	GLMM error distribution (Link)	Biological interpretation
Walking distance	Total distance covered while walking on the petri dish	centimeters (cm)	Gaussian (identity)	Exploration and movement outside and within the hive.
Walking speed	Average walking speed	centimeters per second (cm·s^− 1^)	Gaussian (identity)	Efficient locomotion in-hive and resource collection
Upside down	Lying on its back, moving appendices	Seconds (s)	No observed	Indicate imbalance or motor difficulties
Grooming	Self-cleaning behavior using mouthparts or legs	Seconds (s)	Tweedie (log)	Hygiene, parasite, and particle removal
Wing Fanning	Fluttering wings or attempting to fly	Seconds (s)	Tweedie (log)	Hive ventilation and pheromone dispersal
Food consumption	Syrup consumed by 10 bees over a 24 h period	Weight (g) of syrup	Gaussian (identity)	Metabolic support and energy maintenance
Mortality	Lack of spontaneous movement and response to mechanical stimulation	Number of bees, ranging from 0 to 10 in each cage	Negative binomial (log)	Indicator of lethal effects

### Feeding ability: sucrose consumption and mortality rate

We evaluate the feeding ability of honey bees by measuring the amount of sucrose solution they consume in a controlled environment. After separation and oral administration of the insecticides, bees were placed in circular plastic cages (8.5 cm diameter, 5 cm height), with two lateral openings covered with fabric to allow gas exchanges. Inside each cage, we placed a feeder using a 0.5 mL centrifuge tube (Eppendorf) with the tip cut off, containing 0.5 mL of a 1.7 M sucrose solution. The opening of each feeder was covered with cotton to allow bees access to the solution while preventing spillage. We allocated 10 bees to each plastic cage. Five cages were used per treatment group, totaling 20 cages and 200 bees. Bees analyzed in this phase were not included in the behavioral tests. All cages were maintained in a dark condition in the laboratory, with controlled temperature and humidity for 24 h (25 °C, 65% RH). After this period, the feeders were weighed to quantify sucrose consumption, and the number of dead bees in each cage was recorded.

### Data analysis

Statistical analyses and figures were performed in the R environment (R Core Team 2025). For mortality and food consumption analyses, generalized linear models (GLMs) were fitted using the *glmmTMB* package (Brooks et al. 2017). For the remaining response variables, generalized linear mixed models (GLMMs) were fitted, with treatment groups (Control, DEL, IMI, COM) specified as a fixed effect and experimental block included as a random effect. We describe response variables with error distributions in Table 1. Mixed models were specified according to biological hypotheses and experimental design, and model selection was performed using a backward stepwise procedure based on the Akaike Information Criterion (AIC), evaluating alternative random-effect structures and error distributions to identify the most parsimonious model with adequate fit. Model assumptions were assessed through visual inspection of residuals by comparing observed and expected values, implemented with the *DHARMa* package (Hartig 2024). Planned pairwise contrasts, defined a priori according to the experimental design, were conducted using the *emmeans* package (Lenth 2024) with a Tukey’s correction. Effect sizes, estimated parameters, and associated confidence intervals were extracted using *sjPlot* (Lüdecke 2024) and *ggeffects* (Lüdecke 2018) used to generate model-based plots.

## Results

### Time spent on behaviors: grooming, wing fanning, moving

**Fig. 2:**
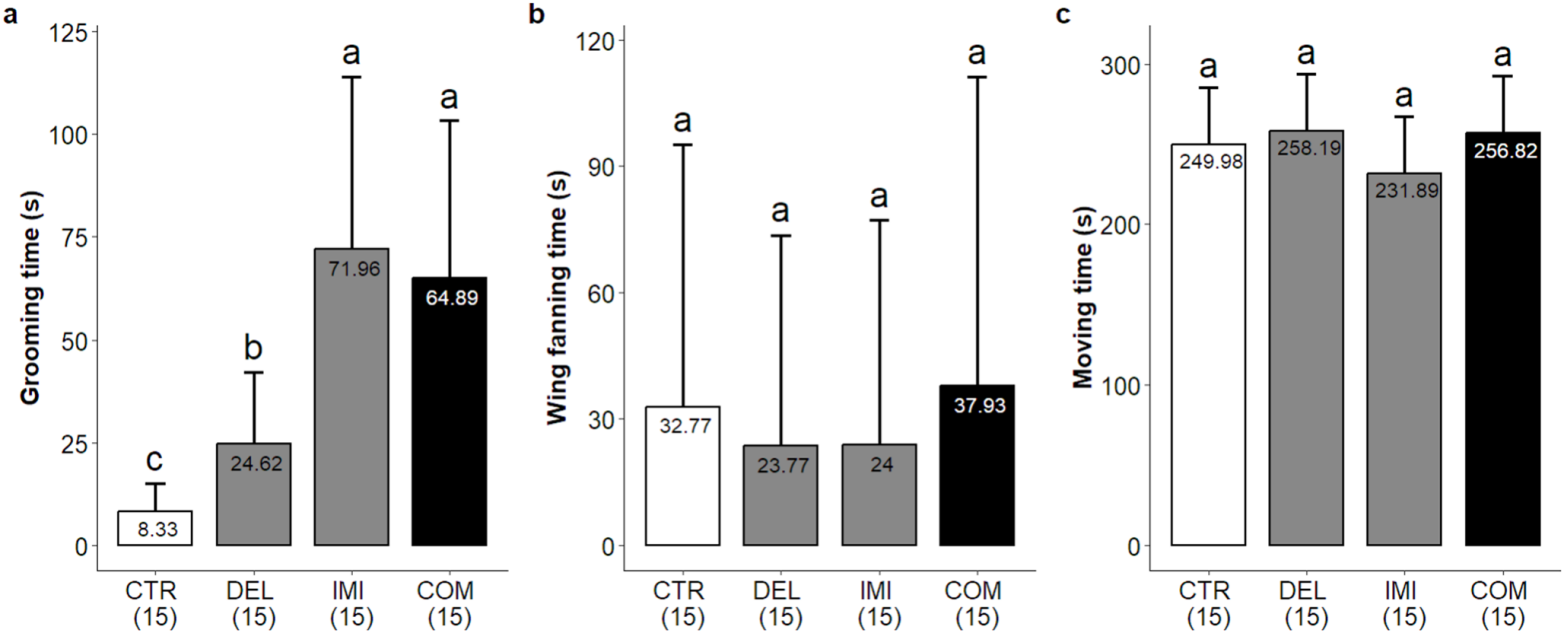
Time spent on each behavioral activity predicted by a GLMM with the following structure: behavioral activity ~ Group + (1 | block). **a** Grooming behavior (s); **b** wing fanning (s); **c** Moving time (s). Bars represent model-predicted means from the fitted model; error bars represent 95% confidence intervals. Different letters above the bars denote significant differences among treatments (pairwise comparisons, *p* < 0.05). CTR = Control; DEL = Deltamethrin; IMI = Imidacloprid; COM = Combination. Sample sizes for each treatment are indicated in parentheses

All insecticides tested, including their combination, altered grooming activity relative to the control group, which spent a mean of 8.33 s (95% CI: 4.57–15.17) in this behavior. Bees exposed to Deltamethrin spent a mean of 24.62 s (95% CI: 14.38–42.17), corresponding to a 2.96-fold increase (95% CI: 1.40–6.23). Imidacloprid and the insecticide combination produced markedly stronger effects, with mean grooming durations of 71.96 s (95% CI: 45.46–113.92) and 64.89 s (95% CI: 40.81–103.17), respectively. These values represent effect sizes of 8.64-fold (95% CI: 4.33–17.24) for Imidacloprid and 7.79-fold (95% CI: 3.92–15.48) for the insecticide combination (Fig. 2a). Pairwise comparisons showed that Imidacloprid elicited 2.92 times more grooming than Deltamethrin (95% CI: 1.54–5.53), with no significant difference relative to the combination treatment (95% CI: 0.62–1.98).

Statistical analysis revealed that none of the insecticide treatments had a significant effect on the time spent on wing fanning behavior (*p* > 0.05; Fig. 2b). Exposure to the insecticides caused no significant effect on bees’ time moving (Fig. 2c) for all tested groups (*p* > 0.05) and the behavior of lying on its back was not observed in any group. No mortality was recorded among the groups during the 30 min metabolization or the behavioral trials.

### Locomotion (walking distance and walking speed)

**Fig. 3:**
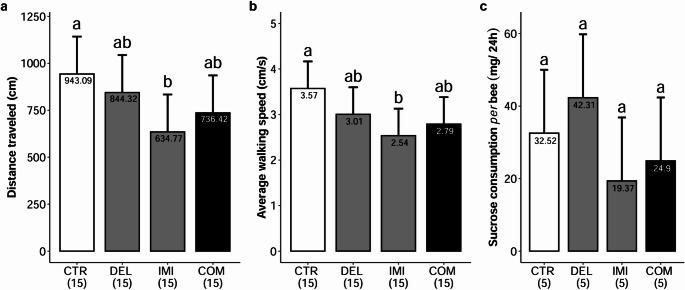
**a** Distance traveled in centimeters (cm) by honey bees across experimental groups GLMM predicted (distance traveled ~ Group + (1 | block)). **b** average walking honey bee speed in centimeters per second (cm·s^− 1^) predicted by GLMM (average speed ~ Group + (1 | block)) and **c** Predicted syrup consumption per cage (syrup consumption ~ Group). Bars represent model-predicted means from the fitted generalized linear mixed model, with error bars indicating 95% confidence intervals. Different letters above the bars denote significant differences among treatments (pairwise comparisons, *p* < 0.05). CTR = Control; DEL = Deltamethrin; IMI = Imidacloprid; COM = Combination. Sample sizes for each treatment are indicated in parentheses

Although the total time spent in locomotion (Fig. 2c) did not differ between groups, Imidacloprid exposure altered specific aspects of bees’ movement patterns, while Deltamethrin presented no significant effects. Bees of the control group walked a mean of 943.09 centimeters (95% CI: 744.01–1142.17) in 5 min. Imidacloprid treated bees walked 634.77 cm (95% CI: 435.69–833.85), reducing the total distance traveled by -308.21 cm (95% CI: -589.85 – -26.77), suggesting impairments in motor function or in the motivation to explore the environment (Fig. 3a). Exposure to Deltamethrin and the Combination of insecticides did not cause significant effects (*p* > 0.05) on the distances traveled compared to the control group, spending, respectively 844.32 cm (95% CI: 645.25–1043.40) and 736.42 cm (95% CI: 537.34–935.50).

Predicted values of walking speed indicate that the Imidacloprid-exposed group showed reduced locomotion speed, with a mean of 2.54 cm·s⁻¹ (95% CI: 1.94–3.13 cm·s⁻¹), whereas the Control group reached a mean of 3.57 cm·s⁻¹ (95% CI: 2.98–4.17 cm·s⁻¹) (Fig. 3b**)**. Imidacloprid caused a walking speed reduction of -1.04 cm·s^− 1^ (95% CI: -1.88 – -0.20 cm·s⁻¹). Walking speed was also reduced by the Combination of the insecticides, with a mean of 2.79 cm·s^− 1^ (95% CI: 2.19–3.38 cm·s⁻¹) marginally significant (*z* = -1.829, *p* = 0.067), with a reduction of -0.78 cm·s^− 1^ (95% CI: -1.62–0.06) by its Combination with Deltamethrin. Exposure to isolated Deltamethrin did not affect the mean speed of the bees, being 3.01 cm·s⁻¹ (95% CI: 2.41–3.60 cm·s^−^¹).

### Feeding ability and mortality

Analysis of mean syrup consumption per bee revealed a reliable estimate for the control group (*p* < 0.001), indicating that, on average, each bee consumed approximately 32.52 mg·24 h^− 1^ (95% CI: 15.05–50 mg·24 h^− 1^). None of the insecticide treatments caused significant effects on syrup consumption, compared to the control (Fig. 3c): DEL: 42.31 mg·24 h^− 1^ (95% CI: 24.83–59.78); IMI: 19.37 mg·24 h^− 1^ (1.89–36.84); COM: 24.90 mg·24 h^− 1^ (7.42–42.37). Beyond that, exposure to insecticides did not alter the mortality in cages over a 24 h period (*p* > 0.05), suggesting a sublethal exposure up to a period of 24 h. Additional data relating to all analyses and models are provided in Online Resource 1.

## Discussion

Our results demonstrate that sublethal exposure to Imidacloprid consistently impaired honey bee behaviors, reducing walking distance and speed, and strongly increasing grooming activity. In contrast, Deltamethrin alone did not affect locomotion parameters, altering only grooming behavior, suggesting a different spectrum and degree of disruption. Interestingly, the combination of both insecticides did not intensify the toxic outcomes; instead, it resulted in intermediate or control-like responses in locomotion performance, and for grooming, activity similar to that caused by Imidacloprid alone. This pattern suggests a non-additive interaction between Imidacloprid and Deltamethrin. Such effects oppose the more common expectation of synergism or additive interaction in pesticide mixtures (Sgolastra et al. 2017; Tosi et al. 2022; Sancho et al. 2025) and suggest that co-exposure may lead to compensatory or interfering physiological responses.

The walking speed and distance traveled reductions support the hypothesis that when exposed to sublethal dose levels of Imidacloprid, honey bees become hypoactive (Tomizawa and Casida 2005), possibly as a result of the overstimulation of the central nervous system by a chronic depolarization of postsynaptic neurons (Williamson and Wright 2013). At lower doses, it induces neural hyperexcitation, whereas at higher doses desensitization leads to paralysis (Raisch and Raunser 2023). These changes corroborate the extensive evidence demonstrating neonicotinoid-induced impairments in motor control, spatial navigation, and foraging (Yang et al. 2008; Henry et al. 2012; Williamson and Wright 2013; Di Noi et al. 2021). Similar alterations at ~ 0.5 ng/bee have been reported for honey bee foraging distance, homing, and feeding behavior (Teeters et al. 2012; Lunardi et al. 2017).

The observed reduction in traveled distance is in accordance with the findings of Tosi et al. (2017) regarding altered exploratory behavior under neonicotinoid exposure. This motor impairment likely stems from Imidacloprid disrupting nAChR-mediated sensorimotor integration within thoracic ganglia—nodes crucial for processing mechanosensory inputs and commanding motor units (Burrows 1996; Ritzmann and Büschges 2007). Furthermore, Imidacloprid increases AChE activity in *Apis mellifera* (Boily et al. 2013), in *Helicoverpa armigera* this insecticide impairs mitochondrial function (Nareshkumar et al. 2018), and can alter dopaminergic homeostasis in *Drosophila melanogaster*, a system modulated by nAChRs that regulates motor initiation (Pyakurel et al. 2018; Martelli et al. 2020). The resulting oxidative stress and dopaminergic-AChE dysregulation (Martelli et al. 2020; Boily et al. 2013) provide a coherent mechanistic explanation for the reduced walking observed in honey bees.

Our results indicate no significant locomotor effect caused by Deltamethrin alone, diverging from previous reports describing pyrethroid-induced sublethal locomotor impairments (Charreton et al. 2015; Tosi et al. 2022). Such discrepancies may reflect differences in experimental design, exposure route, dose, or population susceptibility. Notably, Deltamethrin exposure increased grooming behavior, and at an equal dose (2 ng/bee), impaired cognitive performance in the same honey bee population (Graeff-Filho et al. 2025). Consistent evidence indicates that pyrethroids affect neural systems involved in complex behavioral integration (Vandame et al. 1995; Thany et al. 2015; Field et al. 2017; Mackei et al. 2025), reinforcing that locomotion alone may underestimate the spectrum of pyrethroid sublethal toxicity (Barascou et al. 2021; Bartling et al. 2024).

Grooming is an important self-maintenance behavior sensitive to sublethal insecticide exposure (Sakamoto et al. 2022), and its deficits may have negative effects on the control of parasites and pathogens (e.g. *Varroa destructor*, fungal pathogens) within honey bee hives (Zhukovskaya et al. 2013; Russo et al. 2020). Africanized bees display naturally elevated grooming (Morfin et al. 2020), enhancing defense against parasites such as *Varroa destructor* or *Acarapis woodi*. On the other hand, excessive grooming may negatively affect bees by increasing energetic costs and overlapping with other essential behaviors performed inside and outside the hive (Raine 2018). Sakamoto et al. (2022) found that sublethal exposure to Imidacloprid (0.2 ng/bee) reduces the initial time to removal of *Acarapis woodi*. However, our study investigated self-grooming behavior in the absence of external pathogens and under oral exposure to insecticides. In this context, the intensification of grooming behavior may represent an energetically costly stress response with no apparent benefits (Williamson et al. 2014).

The widespread global use of Deltamethrin and Imidacloprid increases the likelihood of simultaneous exposures, as residues of both compounds have been detected in floral resources such as nectar and pollen (Wen et al. 2021). In our study, combined exposure did not result in statistically significant reductions in walking speed or distance relative to single-insecticide treatments, indicating non-additive effects. Grooming activity, where the most prominent effects occurred, followed a similar pattern, with mean values lower than those observed under Imidacloprid alone. Mathematically, these interactions can be described by different models, such as the Highest Single Agent (HSA) model, where the Combination Index (CI) is calculated as the ratio of the highest effect of a single agent to the effect of the combination (Foucquier and Guedj 2015). For grooming behavior, the calculated CI was 1.11 (CI > 1 indicates a lesser effect than the expected additive or individual performance). As the differences of IMI and COM were not statistically supported, this interaction can be defined as non-additive. Partially associated with this, an antagonistic interaction between orally ingested Deltamethrin and Imidacloprid has previously been described for honey bee cognitive functions, with bees performing better in combined treatments than those of isolated exposures for learning and memory tasks (Graeff-Filho et al. 2025). The physiological mechanisms underlying these interactions remain unclear. However, induction of detoxification pathways, particularly those involving cytochrome P450 enzymes, has been proposed as a possible mechanism mediating reduced toxicity in certain insecticide mixtures (Zhu et al. 2017; Zhang et al. 2022).

Beyond grooming, we assessed additional motor and maintenance behaviors to contextualize sublethal insecticide effects. Total movement time, used as a proxy for exploratory activity, was not affected by any treatment. Wing fanning also showed no significant changes, despite its central role in nest homeostasis, including thermoregulation, gas exchange, and pheromone dispersion (Jones and Oldroyd 2006; Peters et al. 2017). Although these endpoints were unaffected under our experimental conditions, they remain ecologically relevant and may be differentially impacted under field scenarios, warranting cautious extrapolation from laboratory assays.

Finally, bearing in mind that our behavioral assessments were conducted under controlled laboratory conditions, combining automated video tracking with manual observation to detect subtle alterations in locomotion and grooming (Teeters et al. 2012), these laboratory assays do not fully capture the complexity of natural or social environments (Barascou et al. 2021). On the other hand, they allow standardized and sensitive detection of behavioral impairments while minimizing confounding environmental variability (Henry et al. 2014). Manual observation remains particularly valuable for behaviors such as grooming, which are not yet reliably quantified through automated approaches alone. Our observations were taken within a defined time frame, allowing detection of acute effects over a 24 h period. Although it is possible that physiological effects persist over longer periods, potentially leading to delayed, cumulative or chronic-exposure lethal and sublethal outcomes (Tosi et al., 2022; Chen et al. 2021). Chronic behavioral and physiological impairments following prolonged neonicotinoid exposure have been reported in several works with honey bees (Chen et al. 2021), bumble bees (*Bombus terrestris*) (Stanley et al. 2015), and even transgenerational effects have been documented in the offspring of *Drosophila melanogaster* exposed to Imidacloprid (Janner et al. 2021).

We suggest that future research should include comparisons between newly emerged workers and foragers, gene expression, and evaluation of pesticide mixtures, including acute and chronic exposure. Our results show that field-realistic intake of Imidacloprid disrupts locomotion and grooming behavior, while Deltamethrin primarily alters grooming, and their combined exposure does not exacerbate these effects. These findings highlight that behavioral impairment can occur independently of mortality and that mixture effects are not necessarily synergistic. Thus, we reinforce the need for regulatory frameworks to include behavioral endpoints (such as locomotion, grooming, and foraging efficiency) alongside cognitive and transcriptional parameters to improve risk assessment (Dicks et al. 2016; Barascou et al. 2021). To better translate laboratory findings to real-world scenarios, field studies remain essential. Based on our evidence, we support Integrated Pest Management (IPM) strategies aiming to reduce dependence on neurotoxic insecticides, promoting alternatives such as biological control agents, selective pesticides, and agroecological practices.

## Supplementary Information

Below is the link to the electronic supplementary material.

Supplementary Material 1

## Data Availability

Raw data, Rscript and statistical outputs are available in the repository: https://github.com/VagnerGraeff-Filho/Graeff-Filho-Apis_behavioral_disruption.
