# Supplementary material for: Behavioral disruption in honey bees (Apis mellifera) exposed to isolated and combined insecticides
Source: Ecotoxicology. 2026 Apr 28;35(5):107. doi: 10.1007/s10646-026-03095-8 (PMC13124802; doi:10.1007/s10646-026-03095-8)
Supplement: Supplementary file 1 — Supplementary Material 1 [file 10646_2026_3095_MOESM1_ESM.pdf]

Document containing data related to statistical analyses for the work: *Behavioral Disruption in Honey Bees (Apis mellifera) Exposed to Isolated and Combined Insecticides* : Ecotoxicology Journal

Vagner Luiz Graeff-Filho[1,2*]; Luiz Ernesto Costa-Schmidt[2,3]; Stéphane Ramos Idalgo[3]; Felipe Diehl[3]; Cristiano Agra Iserhard[2,3]

[1]Programa de Pós-Graduação em Fitossanidade, Universidade Federal de Pelotas (UFPel), Pelotas, Rio Grande do Sul State, Brasil

[2]Programa de Pós-Graduação em Biodiversidade Animal, Universidade Federal de Pelotas (UFPel), Pelotas, Rio Grande do Sul State, Brasil

[3]Departamento de Ecologia, Zoologia e Genética, Universidade Federal de Pelotas (UFPel), Pelotas, Rio Grande do Sul State, Brasil

* Corresponding author: vagner.filho966@gmail.com

1 Walking speed

1.1 Descriptive table with means (cm/s), sample size (n), standard deviation (SD), and standard error (SE) for walking speed.

Group	Mean	n	SD	SE
CTR	3.57	15	1.27	0.329
DEL	3.01	15	1.39	0.360
IMI	2.54	15	1.15	0.298
COM	2.79	15	1.00	0.258

1.2 Model selection tables based on Akaike Information Criteria (AIC) for walking speed

Mod_name	Family	Random	df	AICc	Delta
mod_velocidade.3	gaussian(id)		5	200.5	0.00
mod_velocidade.4	gaussian(id)	(1 | video))	6	203.0	2.42
mod_velocidade.2	negative binomial (lg)		5	215.3	14.77
mod_velocidade.1	negative binomial (lg)		5	215.3	14.77

1.3 Best model residuals: mod_velocidade.3

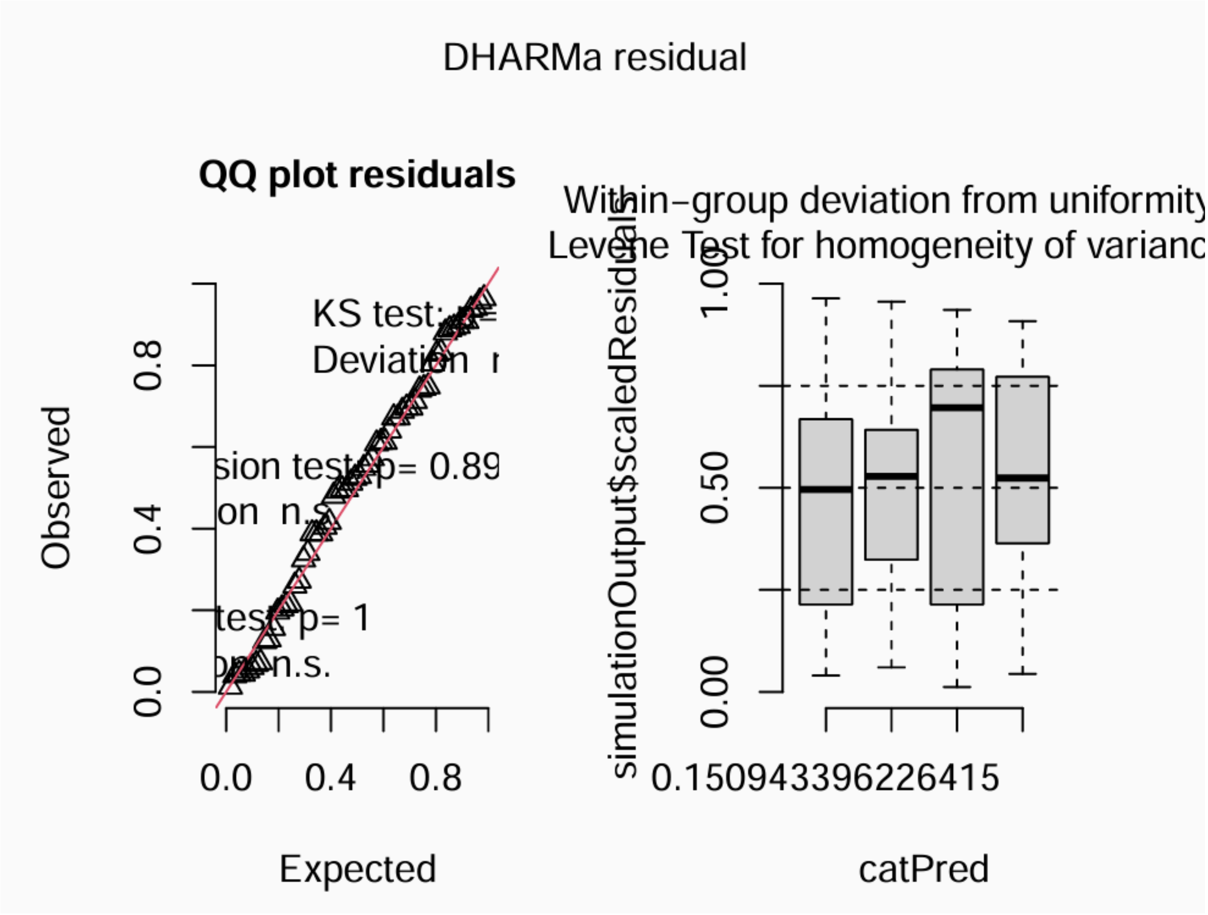

1.4 Best model for walking seed — mod_velocidade.3 (gaussian, identity) — summary

	**Estimate**	**std.error**	**z value**	**p value**
Intercept(CTR)	3.5718	0.3029	11.793	2.10^{-16} ***
DEL	-0.5666	0.4283	-1.323	0.1859
IMI	-1.0357	0.4283	-2.418	0.0156 *
COM	-0.7835	0.4283	-1.829	0.0674 .

2 Traveled distance

2.1 Descriptive table with means, sample size (n), standard deviation (SD), and standard error (SE) for traveled distance (cm).

Group	Mean	n	SD	SE
CTR	943.0859	15	459.5198	118.6475
DEL	844.3245	15	451.5633	116.5936
IMI	634.7754	15	371.0709	95.8101
COM	736.4233	15	332.3832	85.8210

2.2 Model selection tables based on Akaike Information Criteria (AIC) for traveled distance (cm)

Mod_name	Family	Random	df	AICc	Delta
mod_distancia.3	gaussian(id)		5	898.4	0.00
mod_distancia.4	gaussian(id)	(1| Video)	6	900.8	2.47
mod_distancia.2	negative binomial2(lg)		5	909.8	11.44
mod_distancia.1	negative binomial1(log)		5	910.7	12.29

2.3 Best model residuals: mod_distancia.3

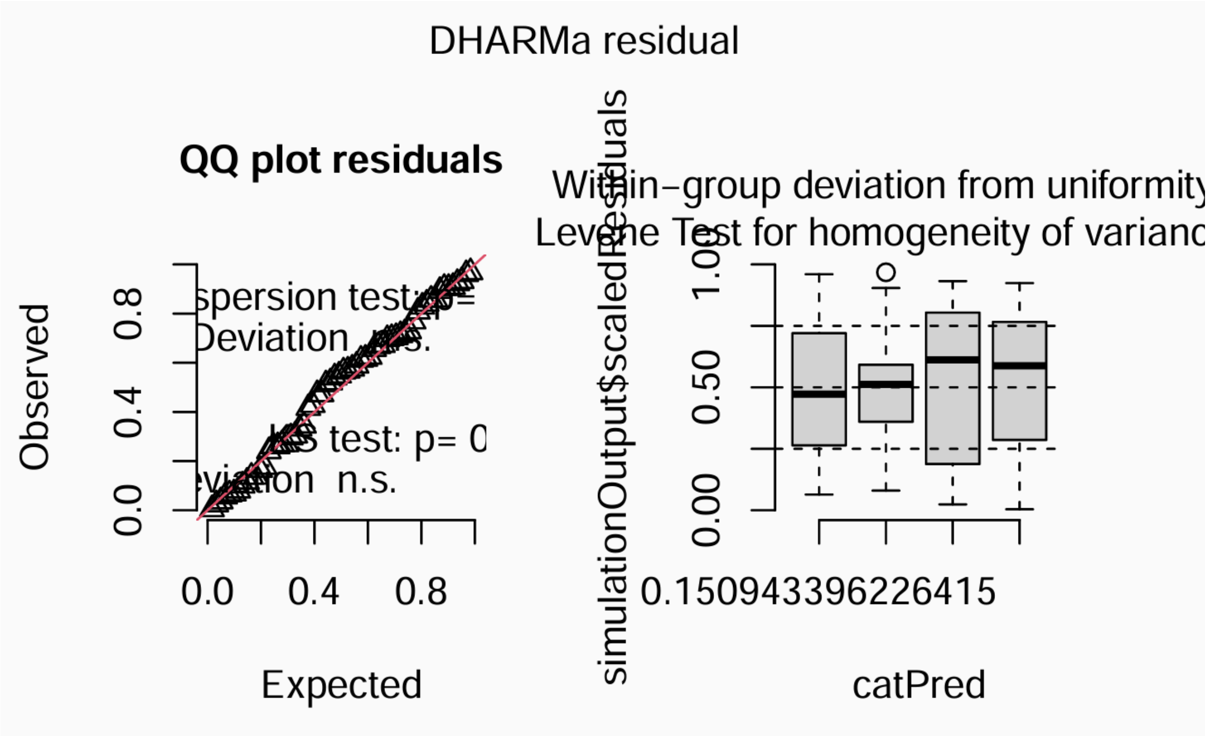

1.4 Best model for walking seed — mod_distancia.3 (gaussian, identity) — summary

	**Estimate**	**std.error**	**z value**	**p value**
Intercept(CTR)	943.09	101.57	9.285	2.10^{-16} ***
DEL	-98.76	143.65	-0.688	0.4917
IMI	-308.31	143.65	-2.146	0.0318 *
COM	-206.66	143.65	-1.439	0.1502

3 Moving time

3.1 Descriptive table with means, sample size (n), standard deviation (SD), and standard error (SE) for moving time (seconds).

Group	Mean	n	SD	SE
CTR	249.9791	15	81.7790	21.1152
DEL	258.1860	15	79.7374	20.5881
IMI	231.8921	15	72.1941	18.6405
COM	256.8207	15	53.0675	13.7020

3.2 Model selection tables based on Akaike Information Criteria (AIC) for time moving (seconds)

Mod_name	Family	Random	df	AICc	Delta
glm_moving.2	gaussian(id)		5	691.4	0.00
glm_moving.3	gaussian(id)	(1| Video)	6	693.9	2.47
glm_moving.1	negative binomial 1(lg)		5	732.2	40.75

3.3 Best model residuals: glm_moving.2

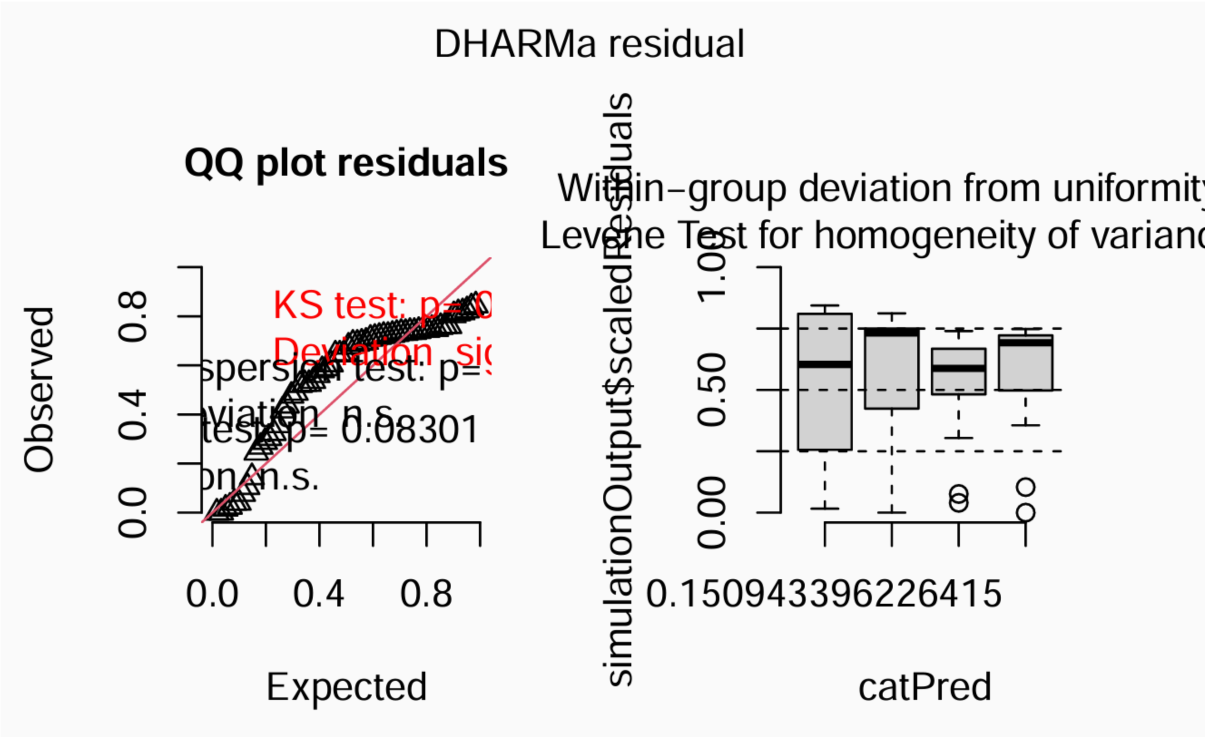

3.4 Best model for walking seed — mod_distancia.3 (gaussian, identity) — summary

	Estimate	std.error	z value	p value
Intercept(CTR)	943.09	101.57	9.285	2.10^{-16} ***
DEL	-98.76	143.65	-0.688	0.4917
IMI	-308.31	143.65	-2.146	0.0318 *
COM	-206.66	143.65	-1.439	0.1502

4 Grooming

4.1 Descriptive table with means, sample size (n), standard deviation (SD), and standard error (SE) for grooming (seconds).

Group	Mean	n	SD	SE
CTR	8.9286	15	11.3033	2.9185
DEL	30.2000	15	58.2990	15.0526
IMI	73.2334	15	56.3139	14.5407
COM	70.8667	15	60.6902	15.6702

4.2 Model selection tables based on Akaike Information Criteria (AIC) for grooming (seconds)

Mod_name	Family	Random	df	AICc	Delta
mod_limpeza.2	tweedie (lg)	(1 | Video)	7	559.0	0.00
mod_limpeza.1	tweedie (lg)		6	559.8	0.78

4.3 Best model residuals: mod_limpeza.2

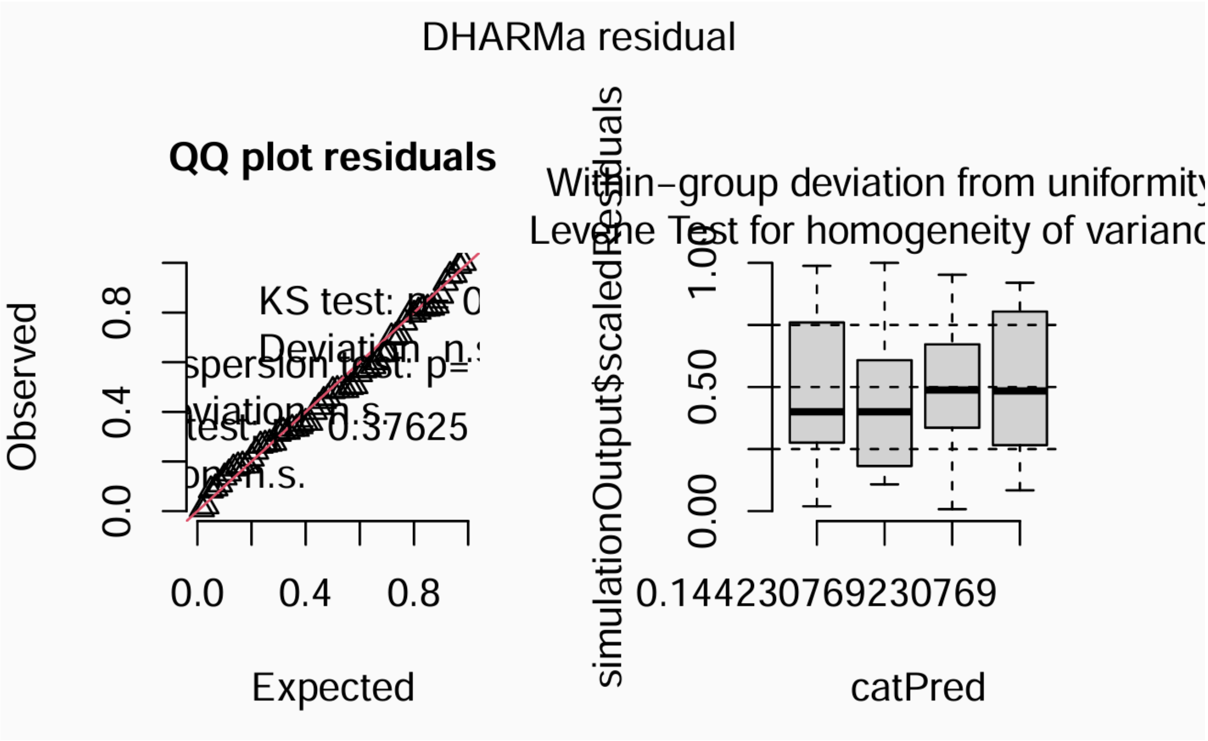

3.4 Best model for grooming — mod_limpeza.2 (tweedie, log) — summary

	**Estimate (log)**	**std.error**	**z value**	**p value**
Intercept(CTR)	2.1195	0.3060	6.927	$4.30.10^{-12}$ ***
DEL	1.0841	0.3802	2.851	0.00435 **
IMI	2.1566	0.3523	6.122	$9.25.10^{-10}$ ***
COM	2.0532	0.3501	5.864	$4.51.10^{-09}$ ***

5 wing fanning

5.1 Descriptive table with means, sample size (n), standard deviation (SD), and standard error (SE) for wing fanning (seconds).

Group	Mean	n	SD	SE
CTR	32.7667	15	59.7575	15.4293
DEL	23.7667	15	76.4415	19.7371
IMI	24.0000	15	57.5837	14.8681
COM	37.9286	15	51.8021	13.3752

5.2 Model selection tables based on Akaike Information Criteria (AIC) for wing fanning (seconds)

Mod_name	Family	Random	df	AICc	Delta
mod_wing.1	tweedie (lg)		6	357.5	0.00
mod_wing.2	tweedia (lg)	(1 | Video)	7	360.0	2.51

5.3 Best model residuals: mod_wing.1

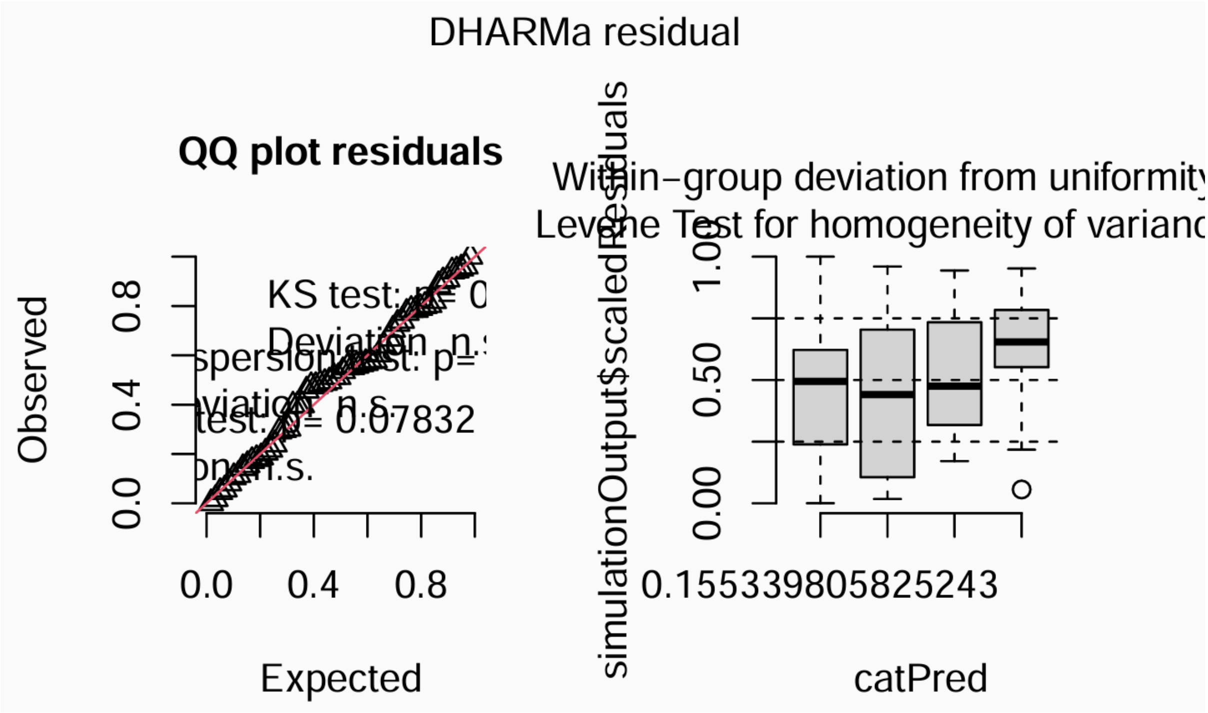

5.4 Best model for wing fanning — mod_wing.1 (tweedie, log) — summary

	**Estimate (log)**	**std.error**	**z value**	**p value**
Intercept(CTR)	3.4894	0.5438	6.416	1.4.10^{-10} ***
DEL	-0.3211	0.7926	-0.405	0.685
IMI	-0.3114	0.8066	-0.386	0.699
COM	0.1463	0.7722	0.189	0.850

6 Syrup consumption

6.1 Descriptive table with means, sample size (n), standard deviation (SD), and standard error (SE) for syrup consumption (mg.24h^{-1}).

Group	Mean	n	SD	SE
CTR	32.5220	5	13.6586	6.1083
DEL	42.3057	5	28.6427	12.8094
IMI	19.3675	5	17.9090	8.0092
COM	24.8963	5	25.6773	11.4833

6.2 Model selection tables based on Akaike Information Criteria (AIC) for syrup consumption (mg.24h^{-1})

Mod_name	Family	Random	df	AICc	Delta
mod_consumo.g	gaussian		5	190.7	0.00
mod_consumo.g.1	gaussian	(1 |rep)	6	194.9	4.12

6.3 Best model residuals: mod_consumo.g

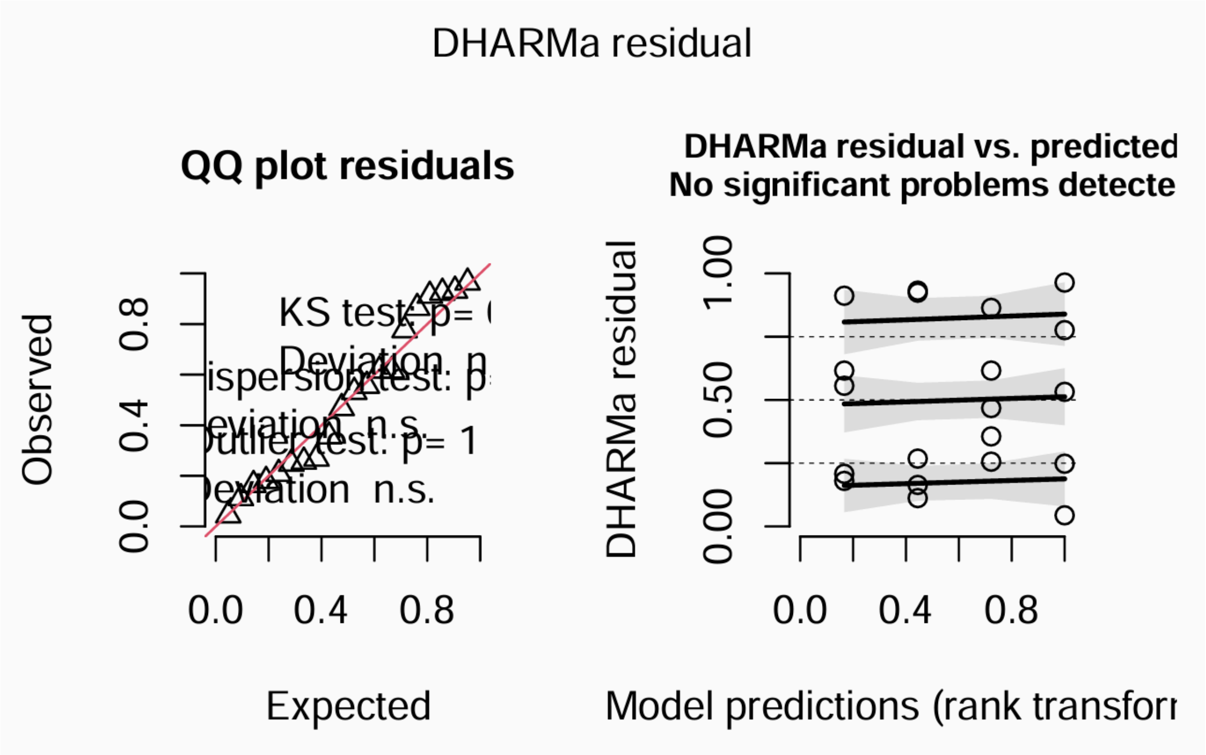

6.4 Best model for syrup consumption — mod_consumo.g (gaussian, identity) — summary

	Estimate (log)	std.error	z value	p value
Intercept(CTR)	32.522	8.915	3.648	0.000264 ***
DEL	9.784	12.608	0.776	0.437752
IMI	-13.155	12.608	-1.043	0.2968
COM	-7.626	12.608	-0.605	0.5453

7 Mortality

7.1 Descriptive table with means, sample size (n), standard deviation (SD), and standard error (SE) for mortality over all laboratory test (count).

Group	Mean	n	SD	SE
CTR	2.8	5	1.4832	0.6633
DEL	3.4	5	1.8166	0.8124
IMI	4.4	5	0.8944	0.4000
COM	3.2	5	2.1679	0.9695

5.2 Model selection tables based on Akaike Information Criteria (AIC) for mortality

Mod_name	Family	Random	df	AICc	Delta
mod_mortalidade.1	gaussian (id)		5	86.8	0.00
mod_mortalidade.2	negative binomial2 (lg)		5	90.0	3.20
mod_mortalidade	gaussian (id)		8	97.9	11.11

4.3 Best model residuals: mod_mortalidade.1

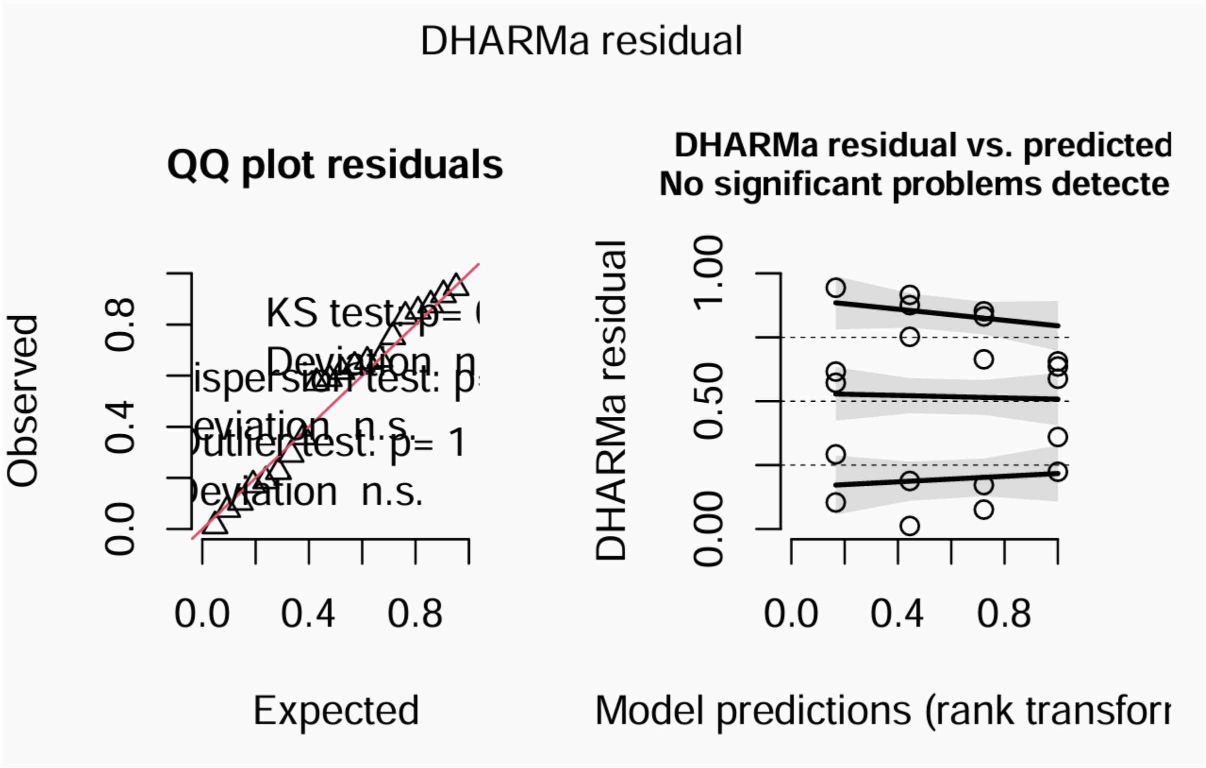

4.4 Best model for syrup consumption — mod_consumo.g (gaussian, identity) — summary

	Estimate (log)	std.error	z value	p value
Intercept(CTR)	2.8000	0.6633	4.221	2.43.10^{-05}
DEL	0.6000	0.9381	0.640	0.5224
IMI	1.6000	0.9381	1.706	0.0881 .
COM	0.4000	0.9381	0.426	0.6698
